# Use of cytobrush for bacteriological and cytological diagnosis of endometritis in mares

**DOI:** 10.14202/vetworld.2024.398-406

**Published:** 2024-02-16

**Authors:** Chiara Del Prete, Francesca Paola Nocera, Giuseppe Piegari, Veronica Palumbo, Luisa De Martino, Natascia Cocchia, Orlando Paciello, Chiara Montano, Maria Pia Pasolini

**Affiliations:** 1 Reproduction Unit, Department of Veterinary Medicine and Animal Productions, University of Naples Federico II, Naples, Italy; 2 Diagnostic Service of Pathology and Animal Health, Department of Veterinary Medicine and Animal Productions, University of Naples Federico II, Naples, Italy; 3 Regional Reference Center for Urban Veterinary Hygiene (CRIUV), Naples, Italy; 4 Surgery Unit, Department of Veterinary Medicine and Animal Productions, University of Naples Federico II, Naples, Italy

**Keywords:** endometritis, field conditions, mare, microbiology brush, uterine sampling

## Abstract

**Background and Aim:**

A combined microbial and cytological examination of uterine samples is the main diagnostic method for endometritis in mares. This study aimed to describe a procedure for using the same uterine cytobrush (CB) for both bacteriological and cytological evaluation.

**Material and Methods:**

The procedure consists of rolling the CB onto a sterilized glass slide immediately after collection and before the transfer into a sterile saline solution. In Experiment 1, a comparison between bacteriological results of the cotton swab (CS) and CB or pellet was made in 10 mares; in Experiment 2, bacteriological and cytological results were compared between different processing methods of CB in 28 mares; in other 6 mares, a CB was processed for cytology only, to investigate the reasons for the low cellularity of the pellet.

**Results:**

The agreement between culture results from the CB and CS was evaluated, and a comparison between the cytological data obtained by different processing methods of CB was performed. The perfect agreement between the CB and CS microbiological results was found. The described procedure enables useful diagnostic smears for cytology. Moreover, the seeding of both the tip of CB and the saline solution used for the transport produced accurate bacteriological results.

**Conclusion:**

The protocol described in this study for the use of CB for both cytological and bacteriological analysis could be used for the diagnosis of endometritis. To maximize diagnostic sample quality, cytology slides must be prepared with meticulous care in the field to preserve cellular integrity and minimize artifacts.

## Introduction

Inflammation of the endometrium is considered a major cause of failure to conceive and embryonic loss in broodmares [1]. Furthermore, subclinical conditions with hidden clinical signs, such as the absence of intrauterine fluid make the diagnosis challenging [2]. Prompt diagnosis of endometritis and effective treatment can improve the chances of pregnancy [3]. A thorough reproductive history, complete reproductive examination (including transrectal palpation and ultrasonography), and uterine sampling are usually necessary for diagnosing endometritis [4].

Combined microbial and cytological examination of uterine samples is mares’ diagnostic mainstay of infectious endometritis. Cytological or bacteriological analysis alone involves a high incidence of false negatives that result from inadequate sampling [5]. While bacteriological culture of uterine swabs is a routinely employed method to confirm an infection, the presence of bacteria alone does not lead to a diagnosis of endometritis. False-positive cultures are common due to contamination of the sampling instrument from the environment, external genitalia, or vagina [6, 7]. A reliable diagnosis of endometritis can be established when the positive culture is supported by the finding of endometrial inflammation in cytology [8]. The combination of these two techniques has the potential to significantly enhance diagnostic accuracy, proving especially beneficial in specific subclinical situations. [5, 8, 9].

Different methods have been proposed for collecting uterine samples for cytological and bacteriological analysis, including non-guarded and guarded uterine culture swab (CS), cytobrush (CB), cytotape, Knudsen catheters, and uterine lavage samples [5, 10–14]. CBs are easy to use under field conditions and are currently considered the technique of choice for cytological examination of the mare’s uterus [5, 11, 12]. Using a double-guarded cotton swab (CS) to obtain samples from the surface of the endometrium for bacteriological culture is a standard practice. Even if different techniques, such as low-volume flush or endometrial biopsy, have proven to be useful in determining the presence of endometrial disease, they are less frequently used in the field due to their costs, complexity, and/or invasiveness [15–17]. Above all, in the field, diagnostic methods should be sensitive and specific but also simple, time-efficient, and cost-effective.

The significance of the study is to validate the use of a single instrument to perform both bacteriological and cytological analysis as an economical and practical alternative in the field, reducing single-use plastic waste. Although the single collection can be less invasive to the animals, the use of the same CB for cytological and culture specimens may increase the risk of false positive cultures due to contamination.

This study aimed to describe a technique for collecting and processing endometrial samples using a CB for both cytological analysis and microbiological culture in diagnosing endometritis in mares. The agreement of bacteriological results between the CS and CB or pellet was tested, and a comparison of cytological results among different processing methods was performed.

## Materials and Methods

### Ethical approval and Informed consent

The study was performed in line with the general recommendations [European Code of Good Veterinary Practice (FVE)]. According to the European Directive EU/2010/63 and Italian regulations (D.lgs. 26–March 04 2014), the approval of the animal welfare committee of the University of Naples Federico II (OPBA) was not required for the described procedures, which qualified as non-experimental clinical veterinary practices. Written informed consent was obtained from the owner of the animals to publish this paper. The welfare of the animals was of utmost importance, and all procedures were designed to minimize pain and distress to the animals.

### Study period and location

The study was conducted from January 2019 to June 2021 at different stud farms in southern Italy (Campania Region), where mares were housed for artificial insemination with frozen or cooled semen.

### Animals

**Figure-1:**
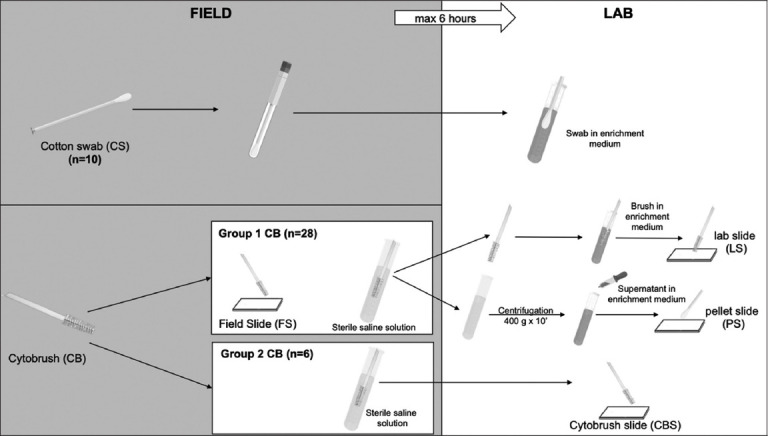
Field and laboratory processing of endometrial samples collected with a cotton swab and cytobrush.

A total of 34 Standardbred mares with a median age of 11 (range 6–17) years were included in the study. Inclusion criteria for the study were the inability to conceive in the previous year or more than three unsuccessful inseminations with a stallion of known fertility during the current breeding season. The experiment was divided into two parts (Experiments 1 and 2), as illustrated schematically in Figure-1 and explained below (experimental design). In Experiment 1, uterine samples from 10 mares were collected, and a comparison between 10 pairwise bacteriological results of the CS and CB or pellet was made. Afterward, a total of 28 Standardbred mares were included in Experiment 2, in which bacteriological and cytological results were compared between different processing methods of CB. All of the patients tolerated the procedures well without any significant complications.

### Experimental design

All mares underwent a complete reproductive evaluation, including transrectal palpation and ultrasonography, evaluation of external genitalia, and inspection of the vagina and cervix by palpation. Clinical examination and transrectal ultrasonography were performed twice per week to assess the estrous stage. All uterine specimens were collected in estrous based on the presence of an ovarian dominant follicle ≥30 mm in diameter, uterine edema (2°–4/5°), and decreased uterine tone [18]. The same experienced veterinarian performed all reproductive evaluations and sampling.

As illustrated schematically in Figure-1, in Experiment 1, to compare bacteriological results in 10 mares, uterine specimens were collected using both double-guarded swab (CS) (Equi-vet, Kruuse, Langeskov, Denmark) and double-guarded CB (Minitube GmbH Tiefenbach, Germany). In these cases, the endometrial swab was performed before CB sampling. In Experiment 2, uterine samples for cytological and bacteriological examinations were collected only by a double-guarded CB (Minitube GmbH Tiefenbach).

For sample collection, the mares were restrained in stocks, the tail was bandaged, and the perineal area was rinsed 3 times with water and povidone-iodine (Betadine^®^, MEDA Pharma S.p.A., Milan, Italy) and then dried with paper towels. Using a long plastic sleeve and sterile gel (ReproJelly, Equi-vet, Kruuse, Langeskov, Denmark), the operator manually guided the covered lubricated double-guarded CB or CS through the vulva, vagina, and cervix and advanced it into the uterus. At this point, the outer tube was retracted, and the CB or CS was positioned in the uterine body in contact with the uterine wall. The CS was rolled in a clockwise direction on the surface of the endometrium for 15 s and then left there for another 15 seconds to collect uterine secretions, whereas the CB was gently rotated alternatively to the right and to the left on the endometrium for 15 s. Then, the CB or CS was retracted into the sheath and removed from the mare.

Immediately, the CS was sealed with sterile caps. Meanwhile, CBs were divided into two groups to evaluate alternative processing conditions. Group 1 CB (n = 28) was rolled onto a sterilized glass slide that was air-dried (field slide [FS]), and then, its tip was cut with sterile scissors and transferred to a small tube containing 4 mL of sterile saline solution. Since the absence of cells for cytological analysis in slides prepared in the laboratory from the smear of the pellet (pellet slide [PS]), an additional experiment on 6 mares (Group 2) was added to investigate whether the processing of CB for the microbiological analysis influences the cellularity of the CB slides. Group 2 CB (n = 6) was directly transferred to a small tube containing 4 mL of sterile saline solution without prior smear on a sterile slide. All specimens were processed at the Microbiological Diagnostic Laboratory of the Department of Veterinary Medicine and Animal Production, University of Naples Federico II (Italy), where they were sent in a cool bag with ice packs within 6 h.

**Figure-2:**
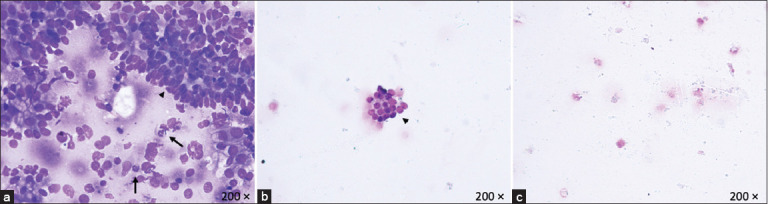
Photomicrographs of equine endometrial cytology smears (Diff-Quick stain, light microscope, 400× magnification) obtained by three different techniques: (a) Rolling a cytobrush onto a sterilized glass slide immediately after collection (field slide [FS]) or (b) after transport into the laboratory in sterile saline solution (lab slide [LS]), or (c) from the pellet after centrifugation of the transport solution (pellet slide [PS]). (a) The cytology smear obtained by FS showed abundant proteinaceous background with good total cellularity, characterized mainly by epithelial cells (arrow) and neutrophilic granulocytes (arrowhead). (b) The cytology smear obtained by LS showed scant total cellularity and proteinaceous background. (c) The cytology smear obtained by PS showed no proteinaceous background and absence of cellularity.

In the laboratory, Group 1 CB was inoculated in a nutrient-rich nonselective enrichment medium for aerobic bacteria and then smeared on a slide for subsequent cytological examination (laboratory slide [LS]). Finally, the CB and the saline solution tubes were centrifuged at 400× *g* 10 min (ALC Centrifuge PK 120, DJB Labcare Limited, United Kingdom). The pellet was first inoculated in broth and then smeared onto a slide (PS; Figure-2).

To further investigate whether the bacteriological procedures impair the quality of cytology smears, Group 2 CB was directly rolled onto a glass slide and air-dried for subsequent cytological examination (CB slide [CBS]).

### Bacteriology

Group 1 CBs, pellets, and CSs were inoculated in a nutrient-rich nonselective enrichment medium for aerobic bacteria, brain-heart infusion broth (BHI), which was then incubated aerobically at 37°C for 24 h (Oxoid, Milan, Italy). After the overnight incubation, the broth culture turbidity was assessed by spectrophotometric reading of the OD value. Turbid BHI tubes were subcultured on Columbia CNA agar (CNA), mannitol salt agar, MacConkey agar, and Sabouraud dextrose agar plates for an additional 24 h at 37°C.

Once bacterial growth was detected, samples were considered positive when one to three different pathogens were recovered. Arisen colonies were first screened by standard and rapid techniques, such as Gram staining, colony morphology and beta-hemolysis on CNA, coagulase, catalase, and oxidase tests. The obtained screening results allowed the choice of specific Api System galleries (Bio Mèrieux, Marcy L’Etoile, France) to identify the isolated strains. The miniaturized strips of biochemical tests were interpreted using an identification database through the online APIWEBÔ service (Bio Mèrieux, Marcy L’Etoile, France). *Escherichia coli* ATCC 25922 and *Streptococcus equi* subspp. *zooepidemicus* ATCC 53698 were included as quality control microorganisms.

### Cytological examination

All slides (FS, LS, PS, and CBS) were stained with Diff-Quick stain (Bio-Optica, Milan, Italy) and evaluated under a light microscope (Nikon Eclipse E600, Florence, Italy) by two independent pathologists (G.P. and O.P.) with a concordance rate of 97%. Each cytology smear was evaluated by observing 10 fields at 200× or 10 fields at 400× magnification (high-power fields [HPFs]). The cells were classified as endometrial epithelial cells, polymorphonucleated cells (PMNs), and other inflammatory cells (eosinophils, lymphocytes, or macrophages). For each field, the following parameters were assessed: background content of the slides (proteinaceous, contaminated with red blood cells, or clear); cell quality (intact, distorted, or fragmented); total cellularity (number of cells/HPF); neutrophils (PMN number/HPF); and the ratio of total cells (mainly uterine epithelial cells) to PMN. The cytological background was graded into the following categories: 1 (absent); 2 (mild proteinaceous background and/or red blood cell contamination); 3 (moderate proteinaceous background and/or red blood cell contamination); and 4 (abundant proteinaceous background and/or red blood cell contamination). Furthermore, based on the PMN/HPF ratio, cytological samples were scored as follows: 1 (no inflammation); 2 (mild inflammation; 0–2 PMNs/HPF); 3 (moderate inflammation; 3–5 PMNs/HPF); and 4 (severe inflammation; >5 PMNs/HPF). Finally, the ratio between total cells and PMNs was graded into the following categories: 1 (vary scant; <0.5%), 2 (scant; 0.5%–5%), 3 (moderate 5%–30%), and 4 (abundant; >30%).

### Statistical analysis

All laboratory data and results were documented on a datasheet using Excel (Microsoft, Redmont, Washinton, USA). Statistical analysis was performed by JMP 12.0 software (SAS Institute Inc, Cary, NC, USA) with significance determined at p < 0.01. Descriptive statistics were used to report bacteriological and cytological results. Cohen’s kappa coefficient (κ) was used to analyze the concordance of positive and negative culture results: the analysis was performed in the first ten mares between the CB or pellet and CS and in the whole sample between the CB and pellet. A κ value of 1 indicates perfect agreement between methods, values of κ > 0.6 indicate good agreement, values between 0.4 and 0.6 indicate moderate agreement, values <0.4 indicate fair agreement, and values <0.2 indicate poor agreement [19]. Furthermore, the qualitative agreement between bacteria isolated by pellets and CBs and those found by conventional culture methods was checked.

The median test compared all samples’ background and inflammatory scores obtained by different slides (FS, LS, PS, and CBS). Comparison tests of all of the cytologic variables considered in the different cytological procedures (FS, LS, and PS) were based on the normality of distribution, as determined by the Shapiro‒Wilk test. Due to the non-normal distribution of data, the Wilcoxon test was applied. The power of each test was determined *post hoc* using G*power 3.1 software (© 2024 Heinrich-Heine-Universität Düsseldorf-https://www.psychologie.hhu.de/arbeitsgruppen/allgemeine-psychologie-und-arbeitspsychologie/gpower), according to Cohen.

## Results

### Bacteriological results

In the first 10 mares, the growth of specific bacteria from either the CB, pellet, or CS was obtained in 4/10 mares (40%), as shown in Table-1. The same bacteria were detected in all pairs of cultures in the CBs, CSs, and pellets; in one case (1/10), a positive culture was isolated from the CS and pellet and not from the CB. The isolated bacteria were *Aerococcus viridans, E. coli, Gardnerella vaginalis, Streptococcus dysgalactiae*, and *Streptococcus* subspp. *zooepidemicus*. The comparison of bacteriological results (positive/negative) between the CS and CB or pellet shows perfect agreement (κ = 1).

**Table-1:** Bacteriological results (positive or negative and isolated bacteria) of the CS, CB, and pellet and their agreement (Cohen’s K) in the first ten mares.

Mare	CS	CB	Pellet	Agreement CS, CB, and pellet	Isolated bacteria
A	-	-	-	=	
B	-	-	-	=	
C	-	-	-	=	
D	+	-	+	≠	*E. coli* + *S. equi* subspp. *zooepidemicus*
E	-	-	-	=	
F	-	-	-	=	
G	+	+	+	=	*G. vaginalis*
H	+	+	+	=	*A. viridans*
I	+	+	+	=	*S. dysgalactiae*
J	-	-	-	=	
Cohen’s K				1	

CS=Cotton swab, CB=Cytobrush, *E. coli=Escherichia coli, S. equi* subspp.=*Streptococcus equi* subspp. *zooepidemicus*, *G. vaginalis=Gardnerella vaginalis,* *A. viridans=Aerococcus viridans,* *S. dysgalactiae=Streptococcus dysgalactiae*

Bacterial cultures were obtained from all 28 mares by inoculation of the CB and pellet in enrichment broth. The bacteriological results (positive/negative) and isolated bacteria are provided in Table-2. In 19 of the 28 mares (68%), the same bacteriological results, positive or negative, were found. The Cohen’s kappa agreement coefficient between the results (positive/negative) obtained after bacteriological cultures of the CB and pellet was moderate (κ = 0.43). Growth of bacteria from either CB or pellets was found in 8/28 samples; a greater number of positive results were obtained by CB cultures (18/28, 64%) than by pellet cultures (11/28). Only in two cases were the isolated bacteria different, as shown in Table-2.

**Table-2:** Bacteriological results (positive or negative and isolated bacteria) of the CB and pellet and their agreement (Cohen’s K) in 28 mares.

Mare	CB	Pellet	Agreement CB versus pellet	Bacteria isolated by CB	Bacteria isolated by Pellet
1	+	+	=	*E. coli* + *S. equi* subspp. *zooepidemicus*	*E. coli* + *S. equi* subspp. *zooepidemicus*
2	-	+	≠		S. lentus
3	+	-	≠	*S. uberis*	
4	+	-	≠	S. dysgalactiae subspp. Equisimilis	
5	+	-	≠	*S. equi* subspp. *zooepidemicus*	
6	+	+	=	*S. xylosus*	*S. xylosus*
7	+	-	≠	*S. equi* subspp. *zooepidemicus*	
8	+	+	=	*S. equi* subspp. *zooepidemicus*	*S. xylosus*
9	+	+	=	*S. uberis*	*S. uberis*
10	+	+	=	*A. urinae*	*A. urinae*
11	-	-	=		
12	-	-	=		
13	+	+	=	*G. vaginalis*	*G. vaginalis*
14	+	+	=	*A. urinae*	*A. urinae*
15	+	-	≠	*S. equi* subspp. *zooepidemicus*	
16	-	-	=		
17	+	-	≠	*K. pneumoniae* subspp. *ozonae*, *S. dysgalactiae*	
18	-	-	=		
19	-	-	=		
20	+	+	=	*A. viridans*	*A. viridans*
21	+	-	≠	*S. equi* subspp. *zooepidemicus*	
22	+	-	≠	*A. viridans*	
23	-	-	=		
24	+	+	=	*E. coli* + *S. equi* subspp. *zooepidemicus*	*E. coli* + *S. equi* subspp. *zooepidemicus*
25	+	+	=	*E. coli*	*E. coli* + S. lentus
26	-	-	=		
27	-	-	=		
28	-	-	=		
Cohen’s K			0.43		

CB=Cytobrush, *S. lentus=Staphylococcus lentus, S. uberis=Streptococcus uberis, S. dysgalactiae=Streptococcus dysgalactiae, S. xylosus=Staphylococcus xylosus, A. urinae=Aerococcus urinae, G. vaginalis=Gardnerella vaginalis, K. pneumonia=Klebsiella pneumonia, Aerococcus viridans=Aerococcus viridans, S. equi* subspp. *zooepidemicus=Streptococcus equi* subspp. *zooepidemicus*

### Cytological examination

PSs reported poor-quality scores; indeed, all of them showed a total absence of cells or low cellularity associated with high numbers of damaged and distorted cells. Regarding FS, LS, and CBS, the number of intact cells and cellularity significantly differed among the three assessed protocols (p < 0.001). Overall, FS showed a higher number of good-quality smears than LS and CBS for both background and inflammatory scores (Table-3). Specifically, all slides prepared by FS showed a mild to abundant proteinaceous background and/or red blood cell contamination, whereas all slides prepared by LS and CBS were clear or had a mild proteinaceous background. No differences were found in the comparison of damaged and distorted cells between different processing methods (FS, LS, and CBS), whereas the total cellularity, intact cells, total cells/HPF, and PMN/uterine epithelial cell ratios were significantly higher in the FS group than in the LS and CBS groups (p < 0.001).

**Table-3:** Cytology results obtained by two different processing methods of the CB in the field (FS) or in the laboratory (LS).

Cytology	Processing method of CB		p-value

	FS	LS	
Background score	2 (1–4)	0 (0–1)	<0.0001
Inflammatory score (1–4)	1.5 (0–4)	0 (0–1)	<0.0001
Total cellularity			<0.0001
Intact cells	70 (15–90)	5 (0–40)	<0.0001
Damaged cells	10 (0–55)	5 (0–90)	0.13
Destroyed cells	15 (10–40)	5 (0–90)	0.28
Total cells/HPF	20 (4–37)	0 (0–7)	<0.0001
PMNs/HPF	2 (0–8)	0	<0.0001
PMNs/uterine epithelial cells	0.11 (0–0.4)	0	<0.0001

p-values are shown for the Wilcoxon test for differences between FS and LS. CB=Cytobrush, FS=Field slide, LS=Lab slide, HPF=High-power fields, PMNs=Polymorphonucleated cells

Based on the ratio between PMNs and HPF, inflammation was classified as absent in 2/28 samples in the FS group and in 27/28 samples in the LS group, mild in 10/28 samples in the FS group, and in 1/28 samples in the LS. Moderate and severe inflammations were observed only in the FS group in eight and four out of 28 samples, respectively. The cytological results, expressed as median (range), of FS and LS are reported in Table-3.

## Discussion

This study explores the viability of a specific protocol that utilizes the same uterine CB for both bacteriological and cytological evaluations in the diagnosis of endometritis in mares.

As demonstrated repeatedly, the combination of cytological analysis with endometrial culture results in increasing the ability to detect endometritis in mares [8, 10, 15]. The use of a single instrument to collect samples for endometrial cytology and microbiological evaluation in mares was previously proposed [12, 20, 21]. Two different devices have been developed for this purpose: the Knudsen catheter and Kalayjian instruments are single-non-guarded swabs for bacteriology equipped with a cup that is used to collect endometrial material for cytological analysis [4, 12, 20]. At present, it is recommended to employ a double-guarded system and adhere to an aseptic sampling approach to prevent contamination from the vagina and clitoral fossa [4]. Recently, the use of CBs to collect endometrial samples suitable for both culture and cytology showed promising results with elevated sensitivity [5, 9]. However, a detailed description of the execution methodology, such as transport conditions and method of inoculation of the CB, was not reported in these studies [5, 9]. Ibrahim *et al*. [14] compared the diagnostic efficacy of endometrial samples collected by CB and cytotape in mares. The CB was rolled onto a sterile glass microscope slide and its tip placed in Stuart medium. Subsequently, the sample was transported a room temperature (20°C) to the laboratory, where it was inserted into a tube containing sterile saline solution. The resulting suspension was vortexed and inoculated into two microbiological plates [14]. In the present study, the authors chose not to immerse the CB in a transport medium but instead directly immersed it in sterile saline. This approach was adopted to prevent contamination of the brush fibers with a gel substance. Following a similar methodology to Ibrahim *et al*. [14], we avoided rubbing the brush directly on the agar plates to mitigate the risk of damaging them with the fibers.

The first concern about the tested procedure was that rolling the brush on the slide in the field could cause bacterial contamination. Thus, Experiment 1 showed that cultures from CB specimens were comparable to those of CSs, processed with a minimal risk of contamination. Although some isolated bacteria may be deemed contaminants or non-pathogens, it is worth noting that all identified bacteria in this study were previously isolated in mares’ uteri [21–24]. Uterine swabs were included as a control for CB samples because they are standard techniques routinely employed for endometrial bacteriological analysis in mares [5]. The perfect concordance in positive and negative results, along with qualitative agreement in bacteriological outcomes between the CB and CS, highlights that meticulous execution of CB sampling and processing can reliably ensure the absence of contamination. In this study, all samplings were performed by the same clinician. It would be interesting to assess whether the risk of contamination is operator-dependent.

The tip of the CB transported in sterile saline was inoculated in an enrichment broth before plating in the agar the day after, as previously described for CSs as an “enrichment methodology” by Nocera *et al*. [24]. Broth enrichment is usually used to improve potential bacterial growth, especially when transport requires a long time [25]. Although the “enrichment methodology” implies a delay of 24 h compared with direct seeding, increased sensitivity has been demonstrated in mares with endometritis [24, 26].

The sensitivity of CB sampling could be increased by the greater exfoliative capacity compared with CS, reaching harbored bacteria deeper in the uterus wall [27–29]. A comparison of swabs and CBs used for vaginal microbiota sampling in women showed higher bacterial loads using the CB, as assessed using quantitative polymerase chain reaction, demonstrating that the CB collects a much higher biomass than the CS and has a greater exfoliative ability [29]. Nevertheless, when the bacterial load was adjusted for the volume of storage media, this difference lost significance and the two methods yielded comparable results when examine the vaginal microbiota composition at the species level [30]. Moreover, in this study, the inoculation of both the CB tip and pellet obtained by the centrifugation of the saline solution, used for transporting the CB into enrichment broth, significantly enhances the sensitivity of bacteriological evaluation. This underscores the importance of recognizing sampling devices and technical procedures as potential sources of variation in the culture results [24].

The CB specimens obtained in the field exhibited superior preservation of both quantity and quality of cells for cytological analysis compared to those obtained from the same brush after transport to the lab in a sterile saline solution. Low-volume flushes, CBs, and CSs have been validated for the cytological evaluation of the endometrium in mares [4, 11, 16, 17]. Moreover, the use of a double-guarded CB has been demonstrated to improve diagnostic accuracy for endometrial cytology in mares and women [5, 8, 11, 12, 21, 31]. Cytological slides prepared immediately after collection had more background, red blood cell contamination, and more intact endometrial and inflammatory cells than LS and PS. The presence of mucus and debris, including degenerated neutrophils, destroyed epithelial cells, and inflammatory residue, in the background of cytological samples may indicate a trace of inflammation and has been consistently associated with positive cultures [16, 17, 31]. The use of the novel cytotape technique demonstrated less blood contamination than the samples collected using a CB [14]. Unfortunately, this instrument is not commercially available but is manually prepared by adapting a double-guarded CS [14].

Loss of cellularity and consequently of the diagnostic sensibility of LS, PS, and CBS compared with FS could be due to transport time, damage caused by centrifugation or failure of the cells to be transferred from the brush to the saline. The centrifugation protocol of this study was based on standard practice in the laboratory, which was also used to recover cells for cytology from uterine low-volume flush [11]. Considering that the same cytological results were observed in LS and CBS, that is, in samples analyzed with and without previous immersion in enrichment broth, the bacteriological procedures can be assumed not to be responsible for different cellularity. In this case, the centrifugation and long-term immersion of the CB into a sterile solution could cause the degradation and rupture of cells [16], even though the chosen transport time (<6 h) and centrifugation methods (400× *g* for 10 min) align with the technique described for low-volume flush [4, 30, 31]. Specifically, samples were processed within 6 h since saline does not preserve bacteria effectively [30]. The CB, composed of polyethylene or nylon without absorptive capabilities [28], employ malleable nylon fibers to prevent cell entrapment when gently rolled onto a slide [28]. In addition, the electrostatic properties of the brush surface were hypothesized to prevent the release of cells in saline, as evidenced by the almost absent cells in the pellet and reduced presence in LS and CBS compared with FS.

For the diagnosis of endometritis, laboratory results need to be interpreted in light of clinical signs [2, 13]. A diagnostic checklist for endometritis, including abnormal clinical findings, modification of a low-volume flush, positive bacterial culture of the pellet, and histologic and cytological evidence of inflammation in the endometrium, has been provided [13]. The advantage of these methods lies in the ability to diagnose endometritis by identifying alterations in two or more parameters of this checklist, thus avoiding endometrial biopsy, the procedure not preferred by owners due to its cost and apparent invasiveness [13].

## Conclusion

The protocol described in this study for using a CB in the cytological and bacteriological analysis of the endometrium is both safe and straightforward, making it practical for use under field conditions. Using a single instrument is cost-effective and contributes to the reduction of single-use plastic waste. However, it is imperative to have a diagnostic cytologic smear to roll the CB onto a glass slide before immersing it in a sterile saline solution. The meticulous processing of CBs under field conditions using sterile slides and a gentle technique in preparing smears is mandatory to reduce the risk of contamination and to allow the collection of more representative samples of the uterine surface. The transport of CB in a saline sterile solution enables the microbiological evaluation of the endometrial sample, and the “enrichment methodology” effectively characterizes the uterine bacterial population by seeding both the tip of CB and the pellet obtained by centrifugation in broth. However, using pellets for cytological analysis is discouraged. The cytological and bacteriological results obtained from a single sampling protocol using CBs should be integrated in an “endometrial checklist” to establish a non-invasive diagnosis of endometritis.

## Authors’ Contributions

CDP: Conceptualization, methodology, writing-original draft, writing-review, and editing. FPN: Data collection and curation; writing-original draft. GP: Data collection and curation; writing-original draft. VP: Data collection and curation. LDM: Methodology. NC: conceptualization, methodology. OP: Methodology. CM: Formal analysis and writing-original draft. MPP: Conceptualization, methodology, writing-original draft, writing-review, and editing. All authors have read, reviewed, and approved the final manuscript.
